# Synapse Plasticity in Motor, Sensory, and Limbo-Prefrontal Cortex Areas as Measured by Degrading Axon Terminals in an Environment Model of Gerbils (*Meriones unguiculatus*)

**DOI:** 10.1155/2009/281561

**Published:** 2009-09-28

**Authors:** Janina Neufeld, Gertraud Teuchert-Noodt, Keren Grafen, York Winter, A. Veronica Witte

**Affiliations:** ^1^Department of Biology and Neuroanatomy, Bielefeld University, 33501, Bielefeld, Germany; ^2^Department of Biology, Humboldt University, Berlin, Germany; ^3^NeuroCure Center of Excellencey, Charité Universitätsmedizin, Berlin, Germany; ^4^Department of Neurology, Charité Universitätsmedizin, Berlin, Germany

## Abstract

Still little is known about naturally occurring synaptogenesis in the adult neocortex and related impacts of epigenetic influences. We therefore investigated (pre)synaptic plasticity in various cortices of adult rodents, visualized by secondary lysosome accumulations (LA) in remodeling axon terminals. Twenty-two male gerbils from either enriched (ER) or impoverished rearing (IR) were used for quantification of silver-stained LA. ER-animals showed rather low LA densities in most primary fields, whereas barrel and secondary/associative cortices exhibited higher densities and layer-specific differences. In IR-animals, these differences were evened out or even inverted. Basic plastic capacities might be linked with remodeling of local intrinsic circuits in the context of cortical map adaptation in both IR- and ER-animals. Frequently described disturbances due to IR in multiple corticocortical and extracortical afferent systems, including the mesocortical dopamine projection, might have led to maladaptations in the plastic capacities of prefronto-limbic areas, as indicated by different LA densities in IR- compared with ER-animals.

## 1. Introduction

Donald O. Hebb postulated in 1949 that synapse plasticity, especially in the neocortex, underlies learning behavior and cognitive flexibility, which has been supported by a number of experimental studies since then [1–3]. Both short-termplasticity and long-term plasticity have been observed, ranging from transient modulations of synaptic effectiveness, such as long-term potentiation and long-term depression [4]*,* to long-lasting, activity-dependent structural modifications of for example, receptor densities [1]. These adaptations result in a strengthening or weakening of synaptic contacts, which in turn leads to degradation [5, 6] or formation [7] of synapses and dendritic spines [8], or even to axonal sprouting or retracting [9].

Particularly hippocampal synaptic plasticity has been thought to be crucially required to constantly adapt to the environment, to learn, and to form memory ([10, 11]*,* for a recent review see, for example, [12, 13]). In the neocortex, use-dependent neuronal changes have been demonstrated, for example, by lesion experiments, revealing at the same time some remarkable differences between functionally diverse cortices. After partial denervation, fairly high plastic adaptation was found in sensory areas of primates [14] and rats [15], whereas low or even no adaptation occurred in motor areas of rats [16, 17]. 

Using noninvasive approaches to correlate learning with structural adaptations, it has been demonstrated that training induces neuronal and synaptic reorganization of cortical maps not only in the visual cortex (e.g., [18]) but also in motor areas [19]. Moreover, it has been assumed that an increase in dendritic length and spine density of pyramidal neurons in somatosensory cortex accounts for better spatial learning of enriched (ER) compared with impoverished-reared (IR) rats [20]. 

Previous studies by our group have revealed that ER leads to an augmentation of prefrontal dopaminergic fibers, which correlated with better learning performance in delayed alternation tasks [21]. Also serotonin, acetylcholine, and GABA were found to be modified by extrinsic activities during brain maturation [22–24], pointing to the crucial role of neurotransmitters in brain plasticity (rev. [22, 25]).

Regarding molecular mechanisms, the main attention has been on the postsynaptic glutamatergic NMDA-receptor, the major excitatory receptor in the mammalian cortex [26–28]. Studying this “learning synapse”, researchers also became aware of the presynaptic role of reciprocal interdependencies between both synaptic elements [29–31].

An exciting approach to take molecular changes within the presynapses as an indirect measure of synaptic remodeling comes from a highly selective, histochemical silver-staining method, which reliably visualizes secondary lysosomal accumulations (LAs) in degrading axon terminals [32]. Several studies have shown that the amount of LA serves as a measure of both primary and reactive degeneration and/or remodeling of presynapses within the brain of rodents and birds [5, 33–37]. 

Further, lifelong synaptic remodeling measured by LA in the dentate gyrus [38–40] correlated significantly with the system-immanent neurogenesis of gerbils [39–42], and hippocampal neurogenesis and synaptogenesis appeared to be crucially affected by environmental factors [39, 43, 44]. Although less is known about neocortical synaptogenesis, the limbo-prefrontal system might offer higher plastic capacities compared with other cortices [45]. To test this assumption, the present study investigated synapse plasticity in functionally diverse neocortical areas in IR and ER gerbils using silver staining of LA.

## 2. Materials and Methods

In total, 22 male gerbils (*Meriones unguiculatus)* were used for quantitative analysis of LA in the neocortex. Gerbils were chosen because of their rich behavioral spectrum, including complex social interactions [46] and their small genetic variability [47]. To evaluate potential effects of early postnatal interventions on cortical plasticity, animals were kept under either enriched rearing (ER, *n* = 11) or impoverished rearing conditions (IR, *n* = 11). ER-animals were bred and reared along with their siblings in 100 × 100 cm large compounds. Therein, wooden facilities to hide and play provided seminatural conditions. IR-animals were bred in standard Makrolon cages type IV. After weaning, on postnatal day (p) 30 they were transferred to standard Makrolon cages type III and kept solitarily but visible and audible to other gerbils. Food and water were provided *ad libitum*. All gerbils were on natural day/night cycles. The experimental procedures were approved by the local committee of animal care in accordance with the guidelines of the European Communities Council Directive.

(Pre)synaptic degradation of axonal terminals as a marker of neuronal plasticity is associated with transient accumulation of secondary lysosomes, multivesicular bodies, and other degrading organelles. For quantification, these lysosomal accumulations (LAs) were visualized by silver impregnation following Gallyas et al. [32]. As previously validated by electron microscopic (EM) studies and ultrathin section analyses, silver impregnation is capable of selectively staining neuronal LAs in remodeling axon terminals [5, 33, 35, 39]. 

On reaching adulthood (p90), animals were transcardially perfused with 5% formaldehyde under deep chloralhydrate anesthesia (1.7 g/kg, i.p.). Brains were dissected and fixed in paraformaldehyde for two weeks at 4°C. Afterwards, frozen brains were bisected, and the left hemispheres were cut in frontal slices of 60 *μ*m thickness on a frigomobile (Reichert-Jung) and retained in phosphate buffer (pH 7). Every second slice from the rostral to the caudal pole of the neocortex was used for staining. Following Gallyas et al. [32], floating slices were prepared in an alkalinic acid (pH 13) containing 9% sodium hydroxide and 1% ammonium nitrate and subsequently silver-impregnated by a silver nitrate solution containing 9% sodium hydroxide, 16% ammonium nitrate, and 50% silver nitrate. The optimum silver concentration was estimated by examining stained test slices by light microscopy. After impregnation, slices were washed three times in changing washing solutions (solution: 30% ethyl alcohol with 0.5 g sodium carbonate mixed with 1% ammonium nitrate). The developer contained 15 mL of 40% formalin and 0.5% citric acid in 1000 mL 10% ethyl alcohol. After another washing, the slices were air-dried, mounted on coated glass slides, dried overnight, and embedded in DePeX (Sigma). 

**Figure 1:**
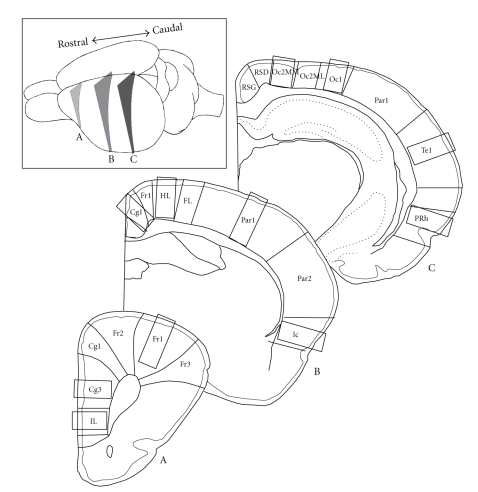
Schematic drawing of the selected cortical areas in which the amount of lysosomal accumulations (LAs) were quantitatively measured. Three coronal sections of the left hemisphere were chosen (left upper corner) including at Bregma +3.2 mm (A) the cingulate cortex area 3 (Cg3), the frontal cortex area 1 (Fr1), and the infralimbic cortex (IL); at Bregma-1.3 mm (B) the cingulate cortex area 1 (Cg1), the hindl**i**mb area (HL), the parietal cortex area 1 (Par1), and the insular cortex (IC); and at Bregma −4.3 mm (C) the occipital cortex area 1 (Oc1) and area 2 mediomedial (Oc2MM), the temporal cortex area 1 (Te1), and the perirhinal cortex (PRh). Rectangles indicate the respective measuring fields.

**Figure 2:**
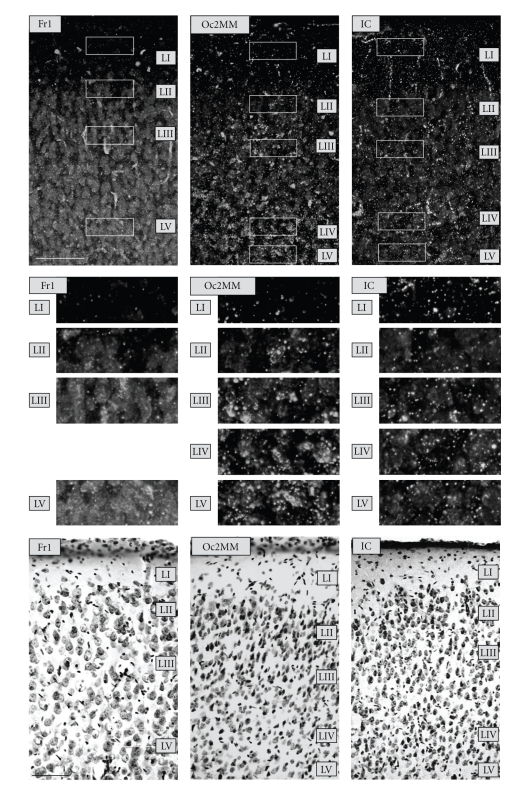
*Top panel.* Degrading axon terminals as indicated by silver-stained granules (light intensities) after Gallyas et al. (1980) [32] throughout the cortical layers of motor (Fr1), secondary sensory (Oc2), and limbic areas (IC) of an enriched-reared gerbil (dark field at 200x magnification). *Middle panel.* For every picture of the Gallyas staining, the marked sections (one per layer) were amplified. Note the distinct distribution of silver-stained granules in the different layers and areas. *Bottom panel.* Congruent sections (Nissl stainings) of the respective cortical areas. Scale bar = 100 *μ*m.

Three different brain sections were viewed in dark field at 125-fold magnification under light microscopy (Olympus, Phillips; see Figure 1). In Section 1 (Bregma +3.2 mm), the following three cortical areas were chosen: the cingulate cortex area 3 (Cg3), the frontal cortex area 1 (Fr1), and the infralimbic cortex (IL). In Section 2 (Bregma –1.3 mm), 4 areas were analyzed: the cingulate cortex area 1 (Cg1), the hindlimb area (HL), the parietal cortex area 1 (Par1), and the insular cortex (IC). In Section 3 (Bregma –4.3 mm), 4 areas were evaluated: the occipital cortex area 1 (Oc1) and area 2 mediomedial (Oc2MM), the temporal cortex area 1 (Te1), and the perirhinal cortex (PRh). Per section and animal, three to five pictures of neighboring slices spanning the whole cortical width were taken by a digital camera (ProgRes 3008mF, Jenoptik, Jena). Three to five slices per section and animal were evaluated to reduce measurement errors, resulting in a total of 9–15 brain slices per animal. Due to artifacts after staining, some areas had to be excluded from evaluation in some animals (ER: Par1, IC, HL, *n* = 4; Cg1, *n* = 5; Fr1, *n* = 6; Cg3, IL, *n* = 7; Te1, *n* = 10; IR: IL, Cg3, *n* = 3; HL, Cg1, Par1, *n* = 4; IC, *n* = 5; Fr1, *n* = 8*)*. Within the respective cortices, well-defined measuring fields were set to estimate the numbers of LAs per cortical layer by a self-developed classification algorithm implemented in MATLAB 6.5 (Figure 2, see also [42]). This algorithm assessed the average number of silver granules (= LA) in every cortical layer. Measuring fields were set in layer I, layer II/III, layer IV (except Fr1, HL, IL, Cg3, and Cg1 due to a relative lack of layer IV in these areas), layer V, and layer VI. They consisted of three adjoining rectangular-shaped subfields (200 × 50 pixels) sized 600 × 50 pixels in total. The height of the measuring field was chosen to fit well into the layers. Analysis was performed under blind conditions.

Statistical analyses were conducted to detect mean differences in LAs (= silver granules) between distinct cortical fields and between different rearing conditions. One-way analyses of variance (ANOVA) with dependent variable “silver granules” and main factor “cortical area” were conducted separately for ER- and IR-animals according to cortical layer. Two-way ANOVA with dependent variable “silver granules” and main factors “cortical area” and “rearing condition” were conducted to detect interaction effects. Subsequent Tukey posthoc testings were used to reveal significant differences. Data are presented as means + standard error (SE). All statistical analyses were computed with Statistica 6 (StatSoft, Tulsa, USA). Levels of significance were set at *P* < .05(*), *P* < .01(**), and *P* < .001(***).

## 3. Results

**Figure 3:**
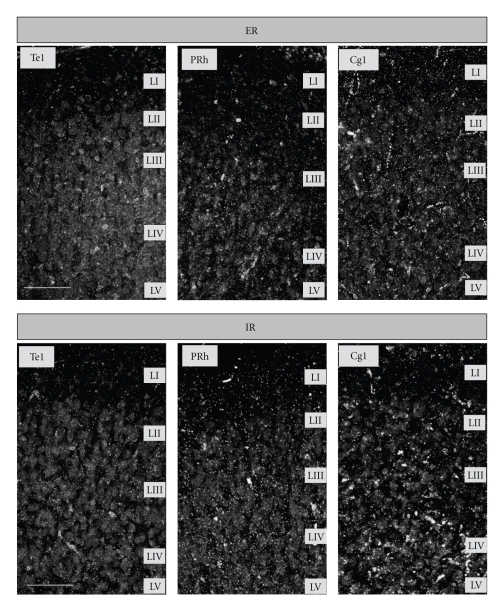
*Top panel.* Degrading axon terminals as indicated by silver-stained granules (light intensities) after Gallyas et al. (1980) [32] throughout primary sensory (Te1) and limbic areas (PRh and Cg1) of an enriched-reared gerbil (dark field at 200x magnification). *Bottom panel.* Congruent sections of the respective cortical areas of an impoverished-reared gerbil. Scale bar = 100 *μ*m.

Dark-field analysis under low magnification indicated a selective distribution of silver-stained granules within the cortex. An intense staining was observed in particular throughout layer I, but also in layers IV and V (Figures 2  and 3). In addition, some remarkable variations were found regarding the staining intensity of different cortical areas. For example, Oc2MM and prefronto-limbic areas were characterized by rather heavy staining, whereas motor fields primarily exhibited weak staining (Figures 2  and 3). 

**Figure 4:**
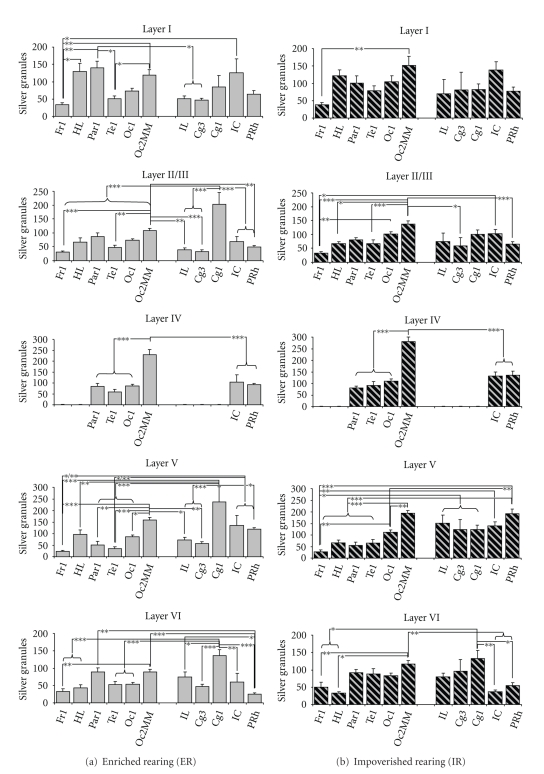
Silver granule densities in the cortical layers of motor (Fr1, HL), sensory (Par1, Te1, Oc1, Oc2MM), and prefronto-limbic areas (IL, Cg3, Cg1, IC, PRh) in enriched- (a) and impoverished-reared (b) gerbils. Note that considerable layer- and area-dependent differences can be seen. Data are given as mean + standard error. Asterisks indicate levels of significance according to posthoc analysis of variance (ANOVA) testings including all areas. *: *P* < .05, **: *P* < .01, ***: *P* < .001. Fr1 : frontal cortex area 1; HL : hindlimb area; Par1 : parietal cortex area 1; Te1 : temporal cortex area 1; Oc1 : occipital area 1; Oc2MM : occipital cortex area 2 mediomedial; IL : infralimbic cortex; Cg3 : cingular cortex area 3; Cg1 : cingular cortex area 1; IC : insular cortex; PRh : perirhinal cortex.

Consistently, one-way ANOVA detected significant effects of “area” on silver granule densities within layers I, II/III, IV, V, and VI in ER-animals when considering all areas (all *F* > 5.3, all *P* < .0001; Table 1 *left*) and in IR-animals (all *F* > 2.8, all *P* < .007; Table 1 *left*). Subsequent posthoc testings revealed significant differences between specific areas in both rearing conditions. In general, hierarchically primary fields were characterized by lower granule density values when compared with secondary/associative fields of the same functional group (motor, sensory, or prefronto-limbic areas; Figure 4). For example, in ER-animals, silver granule densities in the associative sensorimotor area HL were on average twice as high as densities in the primary motor area Fr1 (Figure 4(a)), significant in layer I (*P* = .02). This was also found for the associative sensory area Oc2MM compared with the primary sensory area Te1 in ER-animals, highly significant in the middle layers II/III, IV, and V (all *P* < .002), and significant in outer layer I (*P* = .012). Regarding prefronto-limbic cortices in ER-animals, granule density values of prefrontal area Cg1 were found to be 1.5-fold higher than those of limbic areas, as, for instance, area IL, highly significant in layers II/III and V (all *P* < .001), and significant in layer VI (*P* = .017). A similar situation was evident in IR-animals; albeit area-specific differences were somewhat less explicit (Figure 3), reflected in weaker statistical significance (Figure 4(b); see, e.g., layers I and VI). Another exception emerged in IR-animals regarding prefronto-limbic areas, which will be described herein after in detail.

Significant differences in silver granule densities were also observed between functionally diverse areas in both rearing conditions. On average, density values in sensory areas were twice as high as values in motor fields and *c*. 50% higher than values in prefronto-limbic fields (Figure 4). For example, in ER-animals, IC exhibited significantly higher values compared with Fr1 (Figure 4(a)), significant in layers I and V (*P* < .028). 

**Figure 5:**
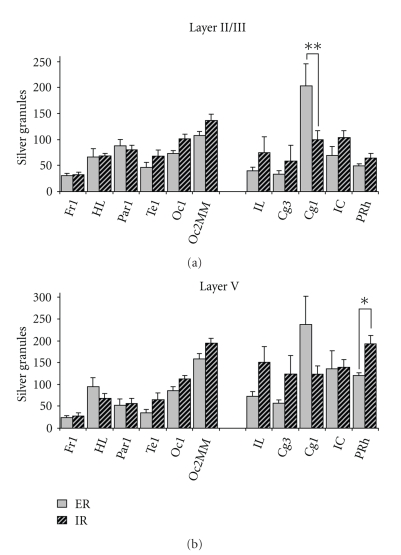
Comparison between silver granule densities of enriched- (ER, uni) and impoverished-reared (IR, striped) gerbils in layer II/III (a) and layer V (b) of motor (Fr1, HL), sensory (Par1, Te1, Oc1, Oc2MM), and prefronto-limbic areas (IL, Cg3, Cg1, IC, PRh). Note higher density values of Cg1 in ER-animals and higher values of PRh in IR-animals. Data are given as mean + standard error. Asterisks indicate levels of significance according to posthoc analysis of variance (ANOVA) testings. *: *P* < .05, **: *P* < .01. For abbreviations see legend to Figure 4.

Some distinct alterations in silver granule densities emerged when comparing the different rearing conditions. In general, values of the different prefronto-limbic areas in IR-animals were somewhat evened out to a similar level in contrast to the well-defined differences seen in ER-animals (Figures 3, 4, and 5). This “flattening” of interareal differences became more obvious when analyzing prefronto-limbic areas separately. In a secondary analysis, we conducted ANOVA with factor “area”, including IL, Cg3, Cg1, IC, and PRh in both ER- and IR-animals. Results showed that significant differences in layers II/III and V, as seen in ER-animals, are completely lost in IR-animals (Table 1, right for *F*-* and P*-*values)*. 

*Moreover*, two-way ANOVA detected a significant effect of the interaction term “group” × “area” in layer II/III (*F*_(10,136)_ = 3.45, *P* < .001) and V (*F*_(10,136)_ = 3.46, *P* < .001). Subsequent posthoc testings revealed significant differences in prefronto-limbic areas. On the one hand, silver granule densities of prefrontal area Cg1 were considerably reduced in IR-animals compared with ER-controls in layer II/III (*P* = .0005; Figure 5(a)) and as trend also in layer V (*P* = .075; Figure 5(b)). In contrast, density values of the limbic area PRh were found to be increased in IR-animals compared with ER-controls in layer V (*P* = .045; Figure 5(b)).

## 4. Discussion

The current study provides for the first time comparative data on spontaneous axon terminal remodeling as indicated by the amount of lysosomal accumulations (LA, [5, 33, 35, 39]) in diverse cortical areas of adult gerbils (*Meriones unguiculatus)*. In animals under enriched rearing (ER) conditions, a rather low synapse turnover was observed in the primary motor, auditory, and visual cortex, whereas rather moderate or exceptionally high LA densities emerged in the hindlimb area, the somatosensory barrel field, an associative visual area, and in prefrontal-limbic subfields. Also, the distribution of LA was found to vary considerably between the different cortical layers (L), with the highest values preferentially in LI, LII/III and LV, yet dependent on the investigated area. Animals from impoverished rearing (IR) conditions exhibited a somewhat similar distribution pattern of LA-indicated axon terminal plasticity. However, compared with ER-animals, layer-specific differences in LA were found to be severely levelled. 

Silver impregnation, after Gallyas et al. [32]*,* has frequently been used to measure synaptic plasticity [5, 33–37, 39, 40, 48]. Quantitative evaluation of LA allows conclusions about the plastic capacities of investigated fields to be made by selectively visualizing degrading (pre)synaptic axon terminals. Thus it indicates one component of synaptic plasticity, namely, synapse turnover or elimination rates (for a detailed dicussion see, e.g., [39])*,* which is not possible with immunohistochemical staining of synaptic markers such as for example, synaptophysin [49]. Another postmortem technique, electron microscopy evaluation of Golgi stainings, is able to more directly detect morphological changes in spines and neurites, but is however restricted to single-neuron analyses (rev. [2, 50]). Using invasive techniques and extensive two-photon laser-scanning microscopy, it is now possible to longitudinally observe changes in spines, axonal branches, and boutons even in vivo [8, 9]*,* but comparison and quantification of plastic capacities between diverse cortical areas are limited. Therefore we used silver staining in the present study. 

Drawing on the present findings, three main aspects will be discussed. First, are area-specific differences comparable to functional and reactive plasticity, as seen for example, after experimental denervation or specific learning tasks? Second, is it possible to identify anatomical pathways which might underlie layer-specific differences in LA-indicated synaptic turnover? Third, to what extent do differences between ER- and IR-animals separate environmental from more intrinsic influences on LA dynamics? Since IR conditions have been shown to induce stereotypies and impair brain functions, we focused on ER-animals, which should exhibit rather intact plastic capacities within the neocortex underlying natural, wildtype-like behaviors. 

Concerning the primary motor field Fr1, synapse turnover as visualized by LA was constantly observed throughout all layers, yet turned out to be extremely low compared with other investigated areas. This is in line with previous studies revealing only restricted lesion-induced plastic capacities in the motor cortex of the rat [16], and reorganization following motor skill learning appearing to occur much more slowly [51]. However, further approaches in the study of motor plasticity indicated that the adult primary motor cortex comprises a dynamic substrate capable of modification and map reorganization in response to motor experience, rather than a static motor control structure [52–54]. This structural dynamic has been proposed as emerging from distributed networks rather than from discrete representations (rev. [54]), thus being part of the local circuit architecture within and between neocortical areas (rev. [55]). Therefore the observed diffuse LA distribution pattern might be explained by axon terminal remodeling of these innercortical network connections. A presumably different axon terminal plasticity occurred in the hindlimb area HL, with significantly increased LA-indicated synapse turnover in LI and moderately increased turnover in LII/III and LV. Given an integrative sensory and motor representation in this area, HL neurons might be exposed to a particularly high level of structural sensory-motor adaptation. This has been observed, for example, during specific motor learning tasks, where both LII/III and LV pyramidal cells underwent large-scale morphological changes for example, in the number of synapses [51, 56, 57]. The exceptionally high amount of LA turnover in LI might be caused by intrinsic ascending recurrent axon collaterals, originating from immediately underlying pyramidal cells and terminating distal tufts of pyramidal apical dendrites [58] or from thalamic projections [59]. Also, commissural connections from the contralateral somatosensory cortex [60] might be a source of higher axon terminal plasticity, as recently indicated using functional magnetic resonance imaging after deafferentation [61].

Considering tactile information processing, the primary somatosensory area Par1 was characterized by selectively increased synaptic turnover in both superficial and deep layers. This is in line with numerous studies reporting enhanced reactive plasticity of the barrel cortex even in adult rodents (rev. [62, 63]). Here, “forward”—as well as “backward”—projections within and between layers III, V, and VI are presumed to sustain local circuit plasticity (rev. [64]), probably contributing to enhanced synaptic turnover. Highest densities of LA, however, were again found in LI. This fairly dynamic remodeling might rather depend on long-range excitatory afferents from projection neurons of premotor and other sensory cortex areas, which are known to selectively form synapses with distal pyramidal dendrites in LI of Par1 [58]. Consistently using two-photon microscopy, De Paola and colleagues found highly plastic terminaux boutons in L1 of rat barrel cortex, formed by long-range axons originating from cortical LVI-pyramidal cells [9]. Thus multiple inputs should be involved in the persistent remodeling process indicated by higher LA densities, to ensure highly adaptive refinements of tactile function.

Low LA densities throughout all cortical layers were found in primary visual and auditory sensory cortex systems, the Oc1 and the Te1. This might depend on species-specific characteristics in the significance and use of the different senses. For example, in adult monkeys a rather prominent axon and synaptic bouton dynamic was found in the visual cortex in vivo [65]. Thus in contrast to primates, gerbils make more use of the sense of touch than of vision and hearing, since the feral wildtype lives as a cave-dweller in the expanses of the Mongolian steppe. Reduced demands on primary cortex processing might also be assumed based on evidence that gerbils are achromatic [66] and that their hearing abilities particularly include ultrasonic waves [67], although at lower frequencies, similar to those of humans [68]. As a source of LA-indicated synapse turnover, one might consider the high amount of intrinsic intra- and interlaminar connections in the rodent primary visual [69] and auditory cortex [70, 71], accomplishing the readaptation of local circuits to match sensory information. Since the adaptive capacity of retino- and tonotopic maps in the adult visual and auditory cortex is however not unimportant for rodents (rev. [72]), the apparent lack of layer-specific LA characteristics in these areas may be considered as one underlying mechanism linking spontaneous synapse plasticity with the plastic ability to reorganize primary cortical maps. A somewhat more complex structural plasticity might be required when considering higher-order processings, as needed for example, for visual attention and object-recognition tasks. Concordantly, the secondary visual area Oc2MM was characterized by elevated synapse turnover in superficial layers and uniquely high values in LIV and LV. Here, connections between area striata and extrastriatal areas might play a crucial role, currently one of the corticocortical pathways best understood [73]. Accordingly, major interactions between lower- and higher-order cortical areas are provided by reciprocal connections between LIV cells of extrastriatal areas and pyramidal cells of the primary visual cortex and other sensory sources [74]. Given these anatomical conditions, it may be assumed that the feedback-recipient cells of area striata might exert the exceptionally dynamic influence on the rewiring of axon terminals in Oc2MM. 

Highest cognitive functions are mediated by an enormous dispersal system including the prefrontal cortex (PFC), which is characterized by special demands on plasticity [75–77]. Interestingly, subfields of the prefrontal-limbic cortex exhibited divergent amounts of synaptic turnover. Remarkably low synapse remodeling indicated by LA was found in infralimbic IL and prelimbic Cg3 areas of the PFC, whereas highly increased levels were observed in the anterior cingulate Cg1 and insular IC cortex. These contrary situations support recent literature on the functional dissociation of the prefrontal-limbic system, demonstrating area-specific and even inverse plastic capacities due to environmental experience, injury, or drug challenge in adult rats [78, 79]. For example, lesioning IL or Cg3 has been shown to impair reversal learning task performance, which is, in contrast, well preserved after pharmacological manipulations of Cg1 [80]. In addition, Cg3, but not IC, appeared to be affected by drug abuse and hormone challenges [81, 82]. The striking synapse plasticity of Cg1 as found in our animals is further in line with electrophysiological ablations demonstrating highly plastic cell responses following drug abuse (rev. [83]). The limbic area PRh, known to mediate between neocortical and limbic areas with important influence on the hippocampus and vice versa [84], therefore exhibited unexpectedly low synapse turnover. This might become significant given that PRh is not the primary, but only one of several parallel routes for sensory information processing to the hippocampus, as found for example, during spatial learning tasks (rev. [85]). By contrast, remarkably high LA densities in the adjacent IC, which is the representative cortex region for limbic and autonomic integration [86], may indeed reflect a persistent plastic adaptation, enforced by various input routes from thalamic, hypothalamic, limbic, and neocortical sources. 

Analysis of layer-specific anatomical characteristics might help to shed further light on the natural prefrontal-limbic synapse plasticity, as indicated by the LA distribution observed in our animals. On one hand, Cg3 and IL share a relatively low structural flexibility [79], with similar low LA densities throughout all cortical layers. Presumably the main constituent of this synapse turnover might be formed by basic recurrent circuits between pyramidal cells and other local pyramidal as well as GABAergic cells [87]. Thus the local networks should be able to provide a spontaneous plasticity, required for example, for attention and response-selection functions in Cg3 [88], as well as visceral functions and fear-related behaviors in IL [89]. Similar basic conditions might be suggested for the PRh interface, which shows a somewhat elevated LA density only around the very distinct population of large LV pyramidal cells, the primary output layer to diverse neocortical systems [85]. On the other hand, a high flexibility of both Cg1 and IC cortex in response to manipulations (e.g., [81, 83]) is in line with the highest LA densities in superficial and/or deep layers, respectively. Characteristically, the IC is subdivided into three subregions, which receive topographically organized afferents from a number of major visceral and limbic sites in the rat brain [86, 90]. Highly specific interconnections between these regions have been suggested to integrate and regulate autonomic and limbic activity [91]. Concerning layer-specific plasticity in Cg1, mainly LIII pyramidal cells are known to play a central role in mediating both between and within cortical areas by intrinsic projections [92]. These long-range projections appear as stripe-like fibers forming reciprocal back-connections to LIII pyramids and to superficial layers [92], which might thus contribute to the uniquely high production of LA in LII/III of Cg1. 

Another source generating elevated anterior cingulate plasticity, for example, within LV and LVI, might be formed by glutamatergic inputs of hippocampal afferents [93, 94]. Thus mainly LV/VI pyramidal cells of Cg1 have been shown to exhibit a selective glutamate receptor-dependent plasticity [95, 96]. According to the present findings, the remodeling of axon terminals as indicated by LA seems to participate in these dynamic processes within Cg1, which serves as one of the central regulatory areas of the prefrontal-hippocampal system [95]. In addition, crucial manipulatory input to Cg1 arises from the mesoprefrontal dopamine (DA) projection, which has been frequently shown to affect for example, working memory and higher cognitive performance [75, 97], and to selectively modulate prefrontal pyramidal cell function [98–100]. Further, our group has described layer-specific DA terminations in LII/III and LV/VI of the mPFC in gerbils [101] that directly correlate with the uniquely high LA values in Cg1 found in the present study. Therefore DA should essentially contribute to the highly dynamic prefrontal plasticity observed both behaviorally and morphologically. 

Further support for this hypothesis comes from previous developmental studies by our group and others. Beside a reduced behavioral and cognitive flexibility linked with impaired prefrontal function, impoverished rearing also caused a suppressive maturation of the mesoprefrontal DA projection [21] and a morphological maladaptation of pyramidal cells in adult rats [102]. Further, we observed dramatically changed maturation patterns in both LII/III and LV/VI prefrontal pyramidal projections to neocortical and limbic regions after IR [103, 104]. Therefore the question arose whether LA-linked mechanisms of plasticity throughout sensory, motor, and associative cortices might be similarly disturbed under the severe DA-reducing condition, namely, IR. In contrast to what might have been expected based on previous experimental studies characterizing experience as a major stimulant of brain structure plasticity [2], our findings revealed, at first glance, relatively few differences in synapse turnover between IR-animals and ER controls. However, some differences became obvious when considering in more detail the area-specific LA distribution in single cortical layers. Most obviously in LI, where area-specific differences in LA densities turned out to be considerably flattened after IR, pointing to a less differentiated plasticity between functionally diverse areas after IR. For example, regarding primary motor/sensory fields, the exceptionally high LA density of Par1, potentially linked with species-specific plastic requirements of this tactile-processing field (see above), is no longer significantly different from values of Fr1 and Te1 in IR-animals. Here, extra-areal corticocortical afferents originating from premotor and sensory areas (rev. [64]) modulate response properties and amplify local circuit activities in target areas [105, 106]. These afferents are assumed to generate the local axon terminal remodeling on distal dendrites of pyramidal cells, which eventually provides the ability to adapt to environmental challenges under normal conditions (cortical maps reorganization see [72]), or after injury, as found in primates [107]. A diminished or less defined LA production after IR in the superficial layer of Par1 might therefore lead to deficits in cortical map reorganization after restricted rearing, as found for example, in rats [108].

Further support for a deprived experience-dependent plasticity in IR gerbils should emerge when comparing LA densities in prefrontal and limbic subfields. Along with again somewhat flattened area-specific differences within single cortical layers, some additional adaptations in response to IR appeared in those areas where LA-indicated plasticity is assumed to depend on challenging information from the environment. On one hand, relatively unchanged LA densities throughout the cortical layers were observed in those regions presumably dominated by local circuitry remodelings, such as IL and Cg3. This was also true for the IC, serving as integrative relay station mainly involved in autonomic processes, which are indeed rather independent of direct environmental influences. However on the other hand, LA values of PRh turned out to be greatly increased exclusively in LV after IR, the layer built by large pyramidal cells (rev. [85]). It has recently been shown that the PRh-hippocampal circuitry, which is somewhat silent in an intact limbic system, becomes functionally more relevant when other paths are lesioned [109]. A somewhat similar situation might be assumed for animals under restricted rearing conditions, namely, that the normally unfavored, reserve-holding PRh-hippocampal path becomes stressed. 

Remarkably, the substantially increased LA density in PRh stands in contrast to a severe decrease in prefrontal Cg1. This dramatic downregulation of LA in all layers of the anterior cingulate cortex might not only disturb plastic capacities of local circuits but also should also interfere with integrative processes via its long-range reciprocal projections to primary and associative cortical areas. Together with the lack of changes in medial and orbital prefrontal subfields, our findings thus support other studies reporting inhomogeneously distributed drug effects on cortical plasticity in rats [81] and area-selective physiological changes following mood disorders in humans as revealed by *post-mortem* and brain imaging studies [110]. In addition, when considering our previous findings of impaired structural DA and pyramidal cell development due to IR in the PFC [21, 103, 104], modulatory DAergic inputs together with the pyramidal glutamate-receptor system (rev. [45]) might indeed contribute to the decrease in LA-indicated synapse plasticity after IR in Cg1. 

Interestingly, in contrast to a suppressive meso-prefrontal maturation, DA fiber densities in limbic structures were found to mature somewhat excessively under IR conditions [111], thus inducing an imbalance of activity flow in limbo-prefrontal circuits. This was thought to provoke discrete malfunctions of neurogenesis and synaptogenesis in the hippocampal dentate gyrus, as found previously by our group after IR (for details, see [39–42]). This imbalance in the DAergic system is also somewhat reflected in the current result of opposite changes in synapse plasticity within areas Cg1 versus PRh after IR. Thus one might further suggest that both limbic and higher-order prefrontal plasticity become impaired by a restricted environment during development, probably due to a maladapted DA maturation. When considering the functional context, one might speculate that under IR, higher-order prefrontal areas become somewhat less activated or utilized in favor of a potentially inappropriately high amount in limbic areas, those areas rather subordinated or controlled by the PFC under normal conditions.

Our data indicate on one hand a rather low natural synapse turnover in primary sensory and motor cortices of adult gerbils, independent of rearing conditions. Presumably, basic intrinsic circuitries involving intra- and interlaminar connections might generate this LA-dependent axon terminal remodeling, providing a rather low structural plasticity, yet sufficient for cortical map reorganization. On the other hand, rather high plastic capacities are thought to include tactile information processing, second-order visual and motor associations, as well as higher-order prefrontal functions. Correspondingly, these cortical systems exhibited higher amounts of LA, especially in those layers involved in extra-areal corticocortical and subcortical circuits. In particular, recurrent circuits between prefrontal and hippocampal pyramidal cells are assumed to play a key role in coordinating and manipulating both local and more distant target regions, by means of the glutamate-receptor-mediated plasticity including DA as essential competitor in the match of balancing systemic adaptation mechanisms. Therefore well-functioning cortical plasticity appears to depend on an optimal level of DA in prefrontal and limbic relay centers. In this context, previous studies by our group have shown that IR-animals are characterized by specific maladaptations, namely, by a suppressive maturation of prefrontal LII/III-pyramidal projections and an excessive maturation of prefrontal LV/VI-pyramidal projections. IR-animals also showed an inverted maturation of DA fibers in both the PFC LII/III and in limbic regions. As indicated by the present data, this might have led to an inversion of plastic capacities between the anterior cingular and the perirhinal cortex after impoverished rearing, again pointing to a severe imbalance of the whole prefrontal-limbic circuitry in these animals. As a consequence, IR-animals would be exposed to dysfunctional cognitive processings throughout life, manifested in severe behavioral deficits [45, 112, 113].

In sum, the present data may offer valuable clues towards a better understanding of synapse plasticity in the neocortex of adult animals. However, further studies are needed to discover and link the underlying mechanisms of both cortical pre- and postsynaptic remodeling, in which obviously the pyramidal cell plays the central role.

## Figures and Tables

**Table 1:** Statistical results from one-way analysis of variance (ANOVA) with main factor “area” in animals under enriched (ER) and impoverished rearing (IR) conditions, conducted for all evaluated areas (left) and prefronto-limbic areas separately (right).

		All areas			Prefronto-limbic areas		
Intervention	layer	*F*	*P*	df	*F*	*P*	df
ER	I	5.36	<.0001	10	2.53	.062	4
	II/III	14.94	<.0001	10	18.35	<.0001	4
	IV	17.05	<.0001	5	0.39	>.1	1
	V	11.94	<.0001	10	7.24	.0004	4
	VI	8.40	<.0001	10	11.26	<.0001	4
							
IR	I	2.77	.007	10	1.44	>.1	4
	II/III	5.74	<.0001	10	1.47	>.1	4
	IV	19.65	<.0001	5	0.021	>.1	1
	V	11.72	<.0001	10	1.69	>.1	4
	VI	4.55	<.0001	10	5.15	.004	4
